# Two important poxviruses that originated in Africa, are spreading rapidly in other continents: why?

**DOI:** 10.1016/j.nmni.2022.101034

**Published:** 2022-09-20

**Authors:** S-L Zhai, M-F Sun, Z-H Xu, C-L Li, G. Wang, C. Zheng, M. Liao

**Affiliations:** 1) Institute of Animal Health, Guangdong Academy of Agricultural Sciences, Key Laboratory of Livestock Disease Prevention of Guangdong Province, Scientific Observation and Experiment Station of Veterinary Drugs and Diagnostic Techniques of Guangdong Province, Ministry of Agriculture and Rural Affairs, Guangzhou, China; 2) The State Key Laboratory of Reproductive Regulation and Breeding of Grassland Livestock, School of Life Sciences, Inner Mongolia University, Hohhot, China; 3) Key Laboratory of Molecular Biology for Infectious Diseases (Ministry of Education), Chongqing Medical University, Chongqing, China; 4) Department of Microbiology, Immunology and Infectious Diseases, University of Calgary, Calgary, Alberta, Canada

**Keywords:** Lumpy skin disease virus, Monkeypox virus, Public health, Pandemic, Poxvirus

Dear Editor,

Poxviruses are the largest DNA viruses and have numerous genus and species. Generally, they can cause typical skin damage to their infected hosts, including humans and many kinds of animals. In human history, through the mass use of the Vaccinia vaccine, smallpox caused by smallpox virus is the only virus that has been wiped out. Theoretically, the poxvirus vaccine can create solid immunity against viral infection. However, two important poxviruses that originated in Africa have been spreading rapidly on other continents in recent years [1,2].

The first one is the lumpy skin disease virus (LSDV) belonging to the Capripoxvirus genus, which mainly affects cattle and occasionally infects non-cattle animals, including eland and giraffes [3]. In addition to contact transmission, insect bites are an important transmission route of LSDV. LSDV was first reported in Zambia, southern Africa, in 1929, then spread in western, eastern, and northern Africa. Before 1988, the prevalence of LSDV only occurred in Africa. But in 1989, LSDV began to enter Israel and Western Asia. Between 1989 and 2014, LSDV was restricted in the Middle East and Western Asia. Since 2015, LSDV has spread to Kazakhstan, Central Asia, and Europe, including Cyprus, Greece, Bulgaria, Serbia, Macedonia, Montenegro, Kosovo, Albania, Georgia, and Russia. In 2019, LSDV entered Xinjiang (bordering Kazakhstan), eastern China. In addition to Africa, Europe, and West Asia, LSDV is widely circulated in East Asia and Southeast Asia, including China, India, Thailand, Vietnam, Nepal, and Bangladesh. A huge threat due to LSDV has been posed in the Asian cattle industry, which has the most cattle in the world [1]. Although there are three kinds of vaccines (one homologous LSDV vaccine and two heterologous vaccines based on sheep poxvirus and goat poxvirus) against LSDV, why is it still causing a pandemic? Based on our knowledge and experience, we believe the main reasons include: 1) lack of good management for border animals; 2) the use of unsafe LSDV live attenuated vaccine leads to the emergence of recombinant strains resulting from live attenuated LSDV vaccine strain and LSDV field strains [4]; 3) lack of a vaccine against LSDV in emerging endemic countries; 4) improper immunization methods; 5) vaccination is difficult to implement in free grazing and wild animals; 6) host and biting insect diversity of LSDV is not conducive to its prevention and control; 7) indifferent awareness of prevention and control of LSDV for farmers and veterinarians, especially in emerging endemic countries; 8) lack of efficient international collaboration.

The second one is the zoonotic monkeypox virus (MPXV), which mainly affects monkeys, Gambian pouched rats, and squirrels, and occasionally infects humans, causing small outbreaks. In addition to fever, headache, muscle aches, and lymphadenopathy, distinctive skin rash is typical. Comparing the above LSDV, MPXV, a relatively young virus, was first found in the Democratic Republic of the Congo (DRC), central Africa (bordering Zambia), in 1970. MPXV, the same as vaccinia virus and smallpox virus, belonged to the *Orthopoxvirus* genus. Genetically, MPXV has two clades: the central African clade and the West African clade. The former could cause higher fatality rates than the latter. However, cross-immune protection existed among them. Due to the previous vaccination of vaccinia, MPXV was endemic in several African countries, including Nigeria, Benin, and Liberia.

With the eradication of the smallpox virus, the cessation of smallpox vaccination in the 1980s lead to a specific immunity decline in humans. In 2017, the big West African MPXV outbreak occurred in Nigeria. Since then, MPXV jumped to Singapore (Southeast Asia), Israel (West Asia), and the United Kingdom (Europe). Incredibly, from 1 January to 1 September 2022, 51,163 confirmed cases of monkeypox and 17 deaths have been reported to WHO from 102 countries/territories/areas in six WHO Regions (Figs. 1A and B) (https://worldhealthorg.shinyapps.io/mpx_global/). The evidence indicates that MPXV is quickly going global [5]. So another “why” should be answered. We believe the main reasons include: 1) frequent foot traffic (such as travelers) and international trade (such as monkeys); 2) novel human-to-human transmission route (especially considerable MSM sexual transmission); 3) disappearance of atypical symptoms (Fig. 1C); 4) lack of a vaccine against MPXV in emerging endemic countries.

**Fig. 1:**
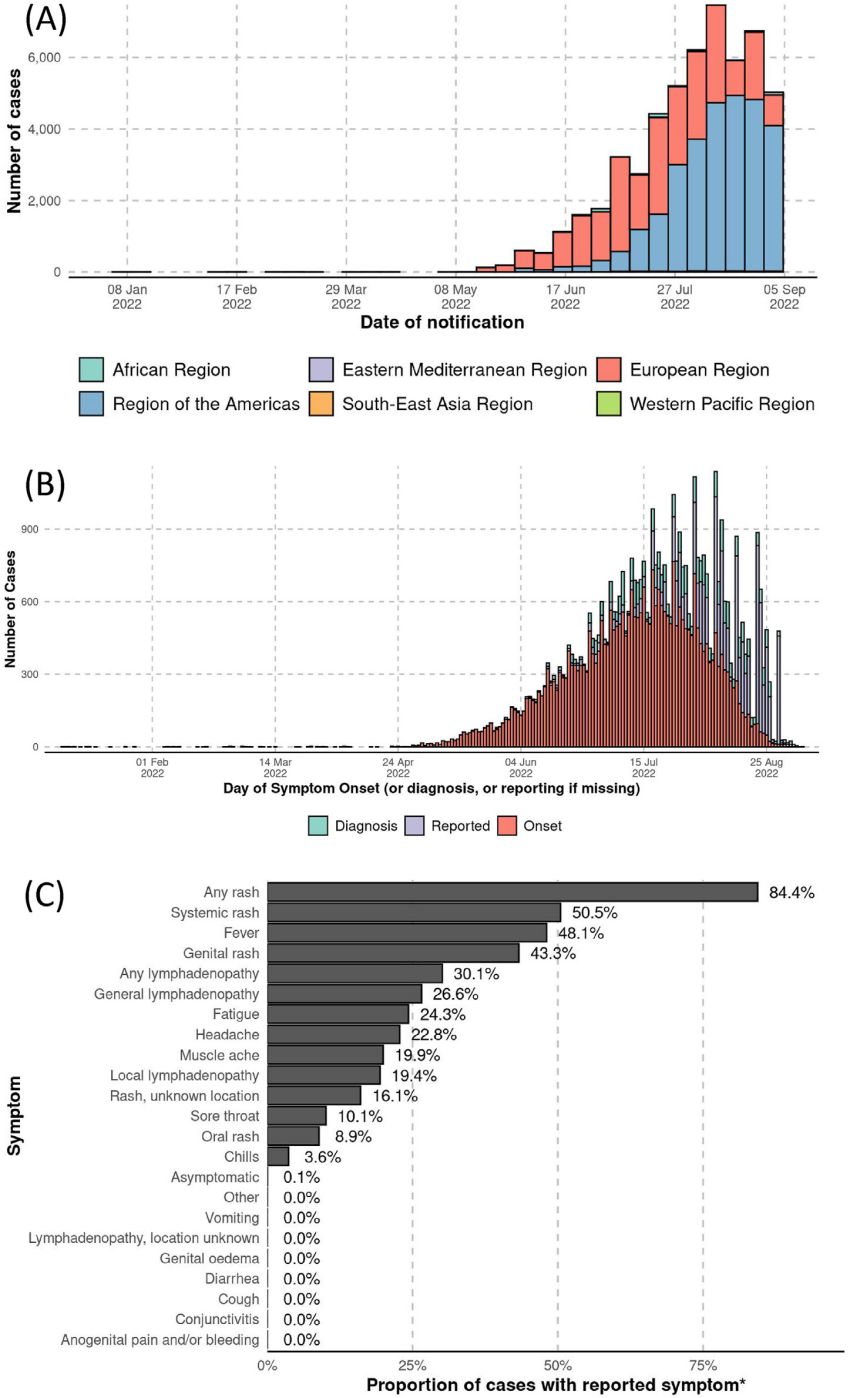
Prevalence and clinical symptoms of monkeypox from WHO since 2022. (A) Prevalence of monkeypox in six WHO Regions since 2022; (B) Daily number of monkeypox cases since 2022; (C) Proportion of monkeypox cases with reported symptom. ∗19,492 cases with at least one reported symptom from a country where at least two unique symptoms reported used as denominator.

LSDV and MPXV can result in huge economic losses and health losses in animals and humans. Under the shadow of the global Covid-19, the control of LSDV and MPXV cannot be ignored. In particular, MPX might be the next pandemic.

## Ethical Approval statement

No ethical approval was required.

## Funding

This study was supported by the following grants: the grant (No. 2021B1212050021 to Z-HX) from the Guangdong Provincial Department of Science and Technology, the grants (Nos. 2021KJ114 and 2021KJ119 to S-LZ) from the Department of Agriculture and Rural Affairs of Guangdong Province, and the grant (No. 201127186621161 to S-LZ) Haojiang District Science and Technology Bureau.

## Author contributions

Shao-Lun Zhai, Ming-Fei Sun, Zhi-Hong Xu are the co-first authors. Shao-Lun Zhai, Ming-Fei Sun, Zhi-Hong Xu collected the data and drafted the paper for the work. Gang Wang, Chun-Ling Li, Chunfu Zheng and Ming Liao review the paper. Shao-Lun Zhai and Zhi-Hong Xu did the financial support, review, and final approval of the paper to be published. All authors read and approved the final manuscript.

## Data availability statement

The data used and analyzed during the current study are available from the corresponding author on reasonable request.

## Conflict of interest

The authors have no competing interests to declare.
